# Supplementary material for: Association between oral microbiome and seven types of cancers in East Asian population: a two-sample Mendelian randomization analysis
Source: Front Mol Biosci. 2023 Nov 21;10:1327893. doi: 10.3389/fmolb.2023.1327893 (PMC10702768; doi:10.3389/fmolb.2023.1327893)
Supplement: Supplementary file 1 [file DataSheet1.ZIP › Supplementary Materials/MR plots for tongue/tongue═╝/Prostate cancer/pheno.787_to_prostate cancer_leave_one_out.pdf]

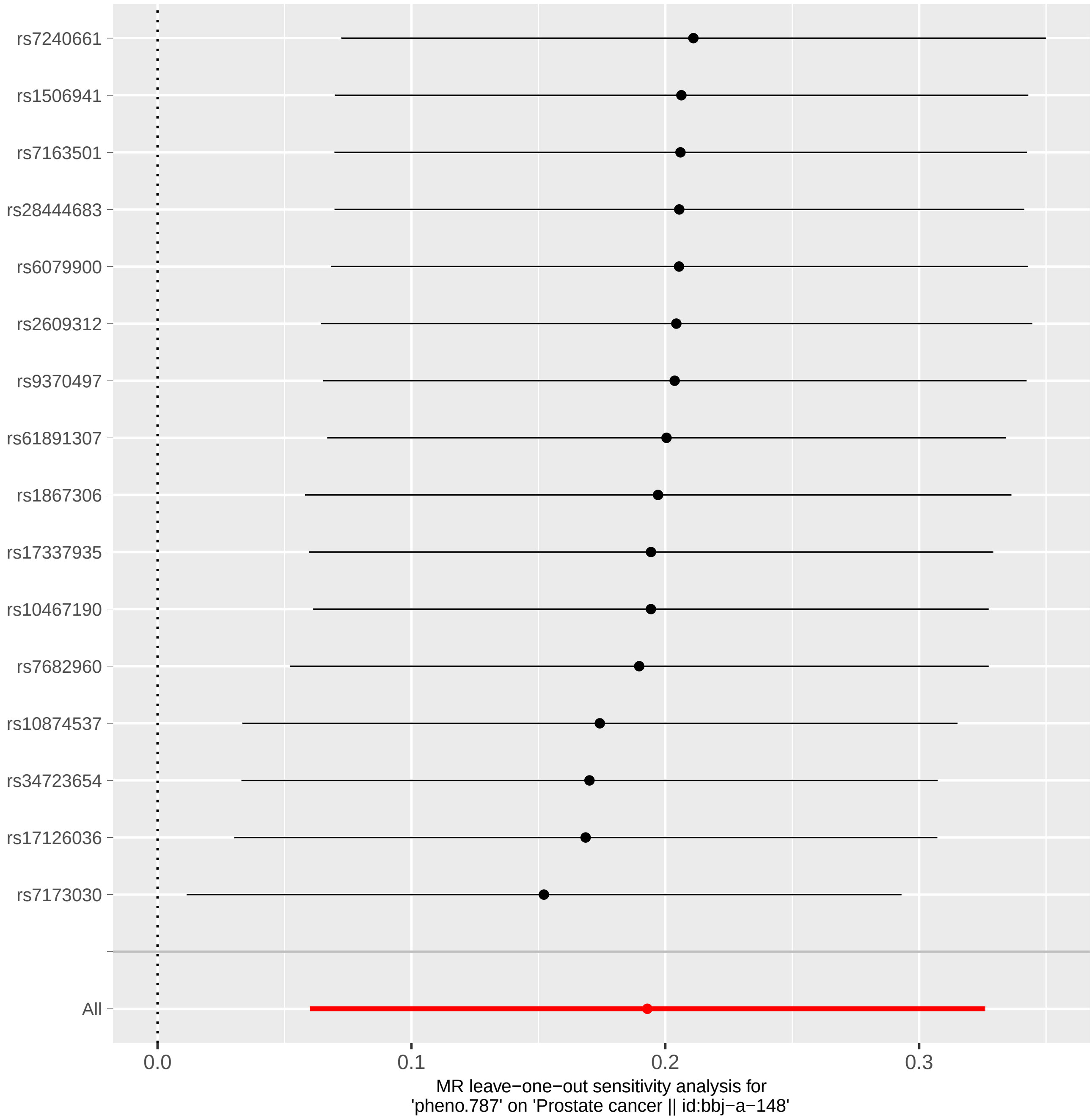

rs7240661
rs1506941
rs7163501
rs28444683
rs6079900
rs2609312
rs9370497
rs61891307
rs1867306
rs17337935
rs10467190
rs7682960
rs10874537
rs34723654
rs17126036
rs7173030
All
0.0
0.1
0.2
0.3
MR leave−one−out sensitivity analysis for
'pheno.787' on 'Prostate cancer || id:bbj−a−148'
